# Genetic Studies on the Inheritance of Storage-Induced Cooking Time in Cowpeas [*Vigna unguiculata* (L.) Walp]

**DOI:** 10.3389/fpls.2020.00444

**Published:** 2020-05-05

**Authors:** Sylvester N. T. T. Addy, Karen A. Cichy, Hans Adu-Dapaah, Isaac K. Asante, Afutu Emmanuel, Samuel K. Offei

**Affiliations:** ^1^Crops Research Institute (CSIR-CRI), Kumasi, Ghana; ^2^USDA-ARS, Department of Plant, Soil and Microbial Sciences, Michigan State University, East Lansing, MI, United States; ^3^West Africa Centre for Crop Improvement, University of Ghana, Accra, Ghana; ^4^Department of Crop Science, School of Agriculture, University of Cape Coast, Cape Coast, Ghana

**Keywords:** heritability, cooking time, cowpea, hard-to-cook, gene action

## Abstract

Cowpeas provide food and income for many small-holder farmers in Africa. Cowpea grains contain substantial quantities of protein, carbohydrates, vitamins, and fiber. In areas where subsistence farming is practiced, cowpea’s protein is cheaper than that obtained from other sources such as fish, meat, poultry or dairy products and combines well with cereal grains in diets. However, long-cooking times, typical of many grain legumes, is a major limitation to the utilization of cowpeas especially among the low-income and growing middle-income population of Africa. Long periods of cooking cowpeas lead to loss of nutrients, loss of useful time and increased greenhouse gas emission through increased burning of firewood. Fast-cooking cowpeas has the potential to deliver highly nutritious food to the hungry within shorter periods, encourage less use of firewood, improve gender equity, increase the consumption of cowpeas, trigger an increase in demand for cowpeas and thus incentivize cowpea production by smallholder farmers in Sub-Saharan Africa. In this study, the inheritance of storage-induced cooking time in cowpeas was investigated. Two sets of bi-parental crosses were conducted involving three cowpea genotypes: CRI-11(1)-1, C9P(B) and TVu7687. Generation means from six generations were used to determine the phenotypic and genotypic variances and coefficients of variation. Broad and narrow sense heritabilities and genetic advance percentage of mean were estimated. Generation mean analysis showed that additive, dominant, additive–additive, additive–dominant, and dominant–dominant gene actions were significant (*p* < 0.001). Fast-cooking trait was dominant over the long-cooking trait. Broad sense heritability for crosses C9P(B) × CRI-11(1)-1 and TVu7687 × CRI-11(1)-1 were 0.94 and 0.99 respectively while narrow sense heritabilities were 0.84 and 0.88 respectively. Genetic advances were 27.09 and 40.40 respectively. High narrow-sense heritabilities and moderate genetic advance for the fast-cooking trait indicated the presence of additive genes in the trait and the possibility of introgressing the trait into farmer-preferred varieties using conventional selection methods. However, due to significant epistatic gene effects observed, effective selection for fast-cooking trait would be appropriate at advanced generations.

## Introduction

Cowpea [*Vigna unguiculata* (L.) Walp] is an edible legume belonging to the family Fabaceae. It is native to Africa and is widely cultivated and consumed in many tropical and subtropical areas of Africa, Latin America, Southeast Asia, and in Southern United States (Appiah et al., 2011). It is commonly known as Southern pea, China pea and Black-eye pea in parts of the world (Olalekan and Bosede, 2010). Cowpeas provide food for humans, livestock and serve as a valuable income-generating commodity for many farmers and grain traders in Ghana and West Africa (Langyintuo et al., 2003; Timko et al., 2008). Its richness in protein makes the crop a source of plant protein (Akpapunam and Sefa-Dedeh, 1997) to people who hardly can afford animal protein from meat, fish, milk, and eggs. As a result, cowpeas contribute immensely to addressing malnutrition among the rural poor in many communities in West Africa. In Ghana, a large section of consumers purchase cowpeas from markets although many cowpea farmers also store quantities for home consumption. Cowpeas are mainly boiled and consumed as whole grains in many parts of West Africa. In Ghana, cowpea is the second most important legume after groundnut. However, its per capita consumption is low (5 kg/year) (Ministry of Food and Agriculture, 2010) compared to common beans, a close relative of cowpeas, with a per capita consumption of up to 34 kg/year in many parts of eastern, central and southern Africa and parts of Latin America (Akibode and Maredia, 2011). One of the major limitations to the increased consumption of cowpeas is their longer cooking times. Long-cooking of cowpeas is therefore a disincentive to their cultivation and consumption. Long periods of cooking cowpeas lead to loss of nutrients, loss of useful time and increased greenhouse gas emission through increased burning of firewood. Fast-cooking cowpeas will help deliver highly nutritious food to the hungry within shorter periods, encourage less use of firewood, improve gender equity, increase the consumption of cowpeas, trigger an increase in demand for cowpeas and thus incentivize cowpea production by smallholder farmers in Sub-Saharan Africa (Addy et al., 2016).

Longer cooking periods render cowpea less economical and acceptable to consumers especially when compared to cereals such as rice which cook in shorter periods. Quaye et al. (2009, 2011) and Egbadzor et al. (2013) reported these conclusions from surveys in Ghana. For this very reason, in many parts of Northern Ghana, cowpeas are popular for this adage: “you don’t wait till you’re hungry before attempting to cook cowpeas.” Typically, cowpeas found on markets in Ghana would have been stored in ambient tropical conditions for between 3 and 6 months (Golob et al., 1998) until premium prices are attained. Storage of cowpeas in these tropical conditions of high temperatures and humidity trigger a grain hardening condition called the ‘hard-to-cook’ phenomenon which leads to increase in cooking duration. This phenomenon is typical of grain legumes (Liu, 1995; Coelho et al., 2007; Nasar-Abbas et al., 2008; Pirhayati et al., 2011). For instance, while common beans in their fresh or non-stored state would generally cook in 22 min, they would later cook in 51 and 140 min after storage in ambient tropical condition for 30 and 60 days respectively (Ribeiro et al., 2005; Coelho et al., 2007). The same phenomenon is experienced in cowpeas but with varying tendencies (Addy, 2016). Thus, increase in cooking time is an indication of the susceptibility of cowpeas to grain hardening. Various procedures including appropriate storage; controlled atmospheres; pretreatments (e.g., steaming, roasting, irradiation, solar drying, microwaving) can be used to prevent or mitigate the development and effect of the hard-to-cook (HTC) phenomenon. However, these procedures do not provide a holistic, economically viable and sustainable solution to this challenge especially in developing countries due to implications on the culture and food systems of its people (Michaels and Stanley, 1988; Reyes-Moreno et al., 1994). Breeding of cowpea varieties less prone to grain hardening or the hard-to-cook state for use in developing countries is by far a more sustainable solution (Reyes-Moreno et al., 1994). A number of studies have been conducted on fast-cooking trait in grain legumes. For instance, studies on cooking time have been conducted for lentils (Erskine et al., 1985); cowpeas (Nielsen et al., 1993) and beans (Elia et al., 1997; Jacinto-Hernandez et al., 2003; Cichy et al., 2015). However, these authors carried out determination of cooking time for grains in their fresh state and not after grains have been kept in storage under tropical conditions. Therefore, a study to determine the mode of inheritance and gene action of this trait would be useful for designing an appropriate breeding program to develop cowpeas possessing resistance to grain hardening or varieties that will remain fast-cooking even after storage.

Generation mean analysis is a simple technique useful for the estimation of gene effects of traits controlled by many genes (Kearsey and Pooni, 2004). The uniqueness of the generation mean model is founded on its ability to estimate additive × additive, dominance × dominance, and additive × dominance gene effects also known as epistatic gene effects (Singh and Singh, 1992). The objectives of this study were to determine the mode of inheritance and gene action for the fast-cooking trait and estimate its genetic advance.

## Materials and Methods

### Cowpea Genotypes

Three cowpea genotypes: CRI-11(1)-1, C9P(B) and TVu7687 were used in this study (Table 1). Genotype CRI-11(1)-1 is an advanced breeding line developed by the Crops Research Institute, Ghana. Genotype C9P(B) is an advanced breeding line obtained from Cinzana Agronomic Research Station, Institut d’Economie Rurale (SRAC-IER), Mali. Seeds of this genotype have a creamy seed coat color, smooth seed coat texture, black helium color and an ovoid seed shape. Genotype TVu7687 is a brown-seeded advanced breeding line developed by the International Institute for Tropical Agriculture (IITA), Nigeria. It has a smooth seed coat texture and a globose seed shape. Genotype CRI-11(1)-1 possesses resistance to storage-induced cooking time while C9P(B) and TVu7687 shows susceptibility to the trait. Its seeds have a rough seed coat texture, a black helium color and a rhomboid seed shape.

**TABLE 1:** Key attributes of cowpea genotypes used in the study.

Genotype	Parent ID	Origin	Seed coat color	Yield potential	Cooking time ‘A’* (minutes)	Cooking time ‘B’* (minutes)
CRI-11(1)-1	P_1_	Ghana	White	High	22	25
C9P(B)	P_2_	Mali	Cream	Moderate	22	200
TVu7687	P_3_	Nigeria	Brown	Moderate	51	303

*‘A’ and ‘B’ - cooking time ‘before’ and ‘after’ storage under high temperatures (30–40°C) and high humidity (60–75%) for 8 months.

### Artificial Hybridization

Artificial hybridization was conducted using the crossing procedure by Rachie et al. (1975) involving two steps: emasculation and pollination. Genotype CRI-11(1)-1 was used as the fast-cooking male parent (P_1_) while C9P(B) and TVu7687 were used as female parents (P_2_ and P_3_) respectively. At podding stage the crossing block was covered with netting to prevent birds from picking the successful pods. Pods that developed from crossing were harvested at physiological maturity. These were dried, threshed, seeds labeled and stored in cold storage. For each cross, seeds for P_1_, P_2_, F_1_, F_2_, BCP_1_, and BCP_2_ generations were obtained. All precautions to ensuring purity and success of crosses were undertaken.

### Field Establishment of Generations

Six generations (P_1_, P_2_, F_1_, F_2_, BC_1_, and BC_2_) obtained for each cross were evaluated at Crops Research Institute, Fumesua in separate randomized complete block designs with three replicates. Experimental units comprised a row each for the non-segregating generations (P_1_, P_2_, F_1_), two rows each for (BC_1_ and BC_2_) and six rows each for the segregating generation (F_2_). Each replicate consisted of 20 plants in one row for each of the parents and F_1_. Thirty (30) plants in three rows each for the backcross generation and 60 plants in 6 rows for the F_2_ population. Two meters long rows with a plant spacing of 20 cm within row and 1.0 m space between rows were used. Two seeds were sown per hill and thinned to one plant per hill. All recommended cultural practices were performed during the growing season. Harvested pods were dried, threshed and kept for cooking quality evaluation.

### Determination of Cooking Time

Harvested seeds were manually sorted to remove extremely small grains, seeds with defective seed coat and extraneous materials such as unhealthy seed, insect infested seed, soil, dust and chaff. Seeds were not treated with storage chemical before or during storage. Sixty (60) seeds were randomly selected from each entry and soaked in 120 ml of 0.1 M acetate buffer (pH 4.0) at 37°C for 7 h and then dried at room temperature for 48 h (Paredes-López et al., 1991; Reyes-Moreno et al., 2001) before cooking time determinations were carried out. Seeds were soaked in (1:4) w/w distilled water for 12 h prior to cooking. Moisture content of seeds ranged from 10–14% as determined with a moisture meter. Data on cooking time were recorded on 10 individual plants from non-segregating populations (P_1_, P_2_, and F_1_) and 20 plants for BC_1_ and BC_2_ and 60 plants for F_2_ population for each replicate. The determination of cooking time was conducted using an experimental pin drop cooker (Wang and Daun, 2005).

### Statistical Analysis

Analyses of variance was done using SAS 9.3 Software (SAS Institute, 2004) to test for significance of difference among generations for cooking time. A significance in differences in generations would indicate the presence of genetic variation. Means were computed for each generation (P_1_, P_2_, F_1_, F_2_, BC_1_, and BC_2_) for each cross over their replications. XLSTAT 2020.1.1 (Addinsoft, 2020) was used to test for normality (Shapiro–Wilk test) for data obtained for F2, BC1, and BC2 generations.

Generation mean was expressed in terms of its genetic effects using the following equation:

Y = m + αa + βd + α*^2^*aa + 2αβad + β*^2^*dd, by Gamble (1962).

where genetic effects were:

Y = the observed mean for generation;

m = mean effect of the F_2_ (i.e., the base population);

a = average additive effects;

d = average dominance effects;

aa = average interactions between additive effects;

dd = average interactions between dominance effects;

ad = additive × dominance gene interaction effects;

α and β = are coefficients for a and d.

Generation mean analysis was performed using Hayman (1958) method. The means for the genetic effects were estimated using the means of each family or generation as:

m = mean of F_2_;

a = BCP_1_- BCP_2_;

d = F_1_-4F_2_-0.5P_1_ -0.5P_2_ + 2BCP_1_ + 2BCP_2_;

aa = 2BCP_1_ + 2BCP_2_ – 4F_2_;

ad = BCP_1_ -0.5P_1_- BCP_2_ + 0.5P_2_;

dd = P_1_ + P_2_ + 2F_1_ + 4F_2_ – 4BCP_1_ – 4BCP_2_.

Variances for the genetic effects were estimated using the variances for each family or generation as:

V_*m*_ = VF_2_;

V_*a*_ = VBP_1_ + VBP_2_;

V_*d*_ = VF_1_ + 16VF_2_ + 0.25VP_1_ + 0.25VP_2_ + 4VBP_1_ + 4VBP_2_;

V_*aa*_ = 4VBP_1_ + 4VBP_2_ + 16VF_2_;

V_*ad*_ = VBP_1_ + 0.25VP_1_ + VBP_2_ + 0.25VP_2_;

V_*dd*_ = VP_1_ + VP_2_ + 4VF_1_ + 16VF_2_ + 16VBP_1_ + 16VBP_2_.

Standard errors for each of the genetic effects were also estimated as the square root of their corresponding variances. Genetic parameters and their variances were tested for significance by calculating ‘t’ tests as ratios of genetic effects to their respective standard errors. Broad-sense (*h_b_*^2^) and narrow-sense heritabilities (*h*_*n*_^2^) were estimated using variances of F_2_ and backcross generations according to Warner (1952).

$$h_{b}^{2}=\frac{\left[2VF_{2}-\left(VP_{1}+VP_{2}+VF_{1}\right)/3\right]\times 100}{VF_{2}}$$

$$h_{n}^{2}=\frac{2VF_{2}-\left(VBCP_{1}+VBCP_{2}\right)\times 100}{2VF_{2}}$$

The degree of dominance (D) which is the deviation from the mid-parent value toward one of the parents was also calculated as the ratio of the dominance to the additive effect according to Falconer and Mackay (1960):

D (degree of dominance) = d/a;

Where $d=F_{1}-\frac{1}{2}\left(P_{1}+P_{2}\right)\text{and}a=\frac{1}{2}\left(P_{1}-P_{2}\right)$

Means, variances, heritabilities, degree of dominance, genotypic and phenotypic coefficient of variation and genetic advance were computed using Microsoft Excel 2013. Genotypic and phenotypic variances and coefficients of variation were computed according to Burton and Devane (1953). Genetic advance to be expected for the selection of five percent of superior progenies was computed according to Robinson et al. (1951) as follows:

Phenotypic variance (δ^2^p) = Var F_2_

Environmental variance (δ^2^e) = Var F_1_

Genotypic variance (δ^2^g) = (δ^2^p – δ^2^e)

Genotypic coefficient of variance (GCV) = $\left(\frac{\sqrt{\delta 2g}}{X}\right)\times 100$

Phenotypic coefficient of variance (GCV) = $\left(\frac{\sqrt{\delta 2g}}{X}\right)\times 100$

Genetic advance (GA) = iδp$h_{b}^{2}$

Where Var F_2_ = variance of F_2_ population;

Var F_1_ = variance of corresponding F_1_ population;

X = general mean of the character;

I = intensity of selection (5% with a value of K being 2.06);

δp = phenotypic standard deviation;

$h_{b}^{2}$ = broad sense heritability.

## Results

### Genetic Variability and Gene Effects

Results of the analysis of variance for the six basic generations (P_1_, P_2_, F_1_, F_2_, BC_1_, and BC_2_) for both crosses are presented (Table 2). The table shows highly significant (*P* < 0.001) differences were detected among generations for both crosses 1 and 2. Differences found among the six generations were observed in the results of population means distribution for cooking time for crosses 1 and 2 (Tables 3, 4). In cross 1, means of generations were in increasing order of P_1_ < BC_1_P_1_ < F_1_ < BC_1_P_2_ < F_2_, < P_2_ (Table 3). However, in cross 2, means of generations were in the increasing order of P_1_ < BC_1_P_3_ < F_1_ < F_2_ < BC_2_P_3_ < P_3_ (Table 4). In both crosses, the means of the F_1_ fell between cooking time values for both parents indicating some level of partial or incomplete dominance. In fact, means of all other generations were less than the higher cooking time parents P_2_ and P_3_. The population mean distributions obtained for both crosses showed that P_1_, BC_1_P_1_ and F_1_ were skewed toward the short cooking time parent. Furthermore, in cross 1, the coefficient of variation for F_2_ was high (1.9%), although lower than coefficients of variation for F_1_ and BC_1_P_1_ (Table 3). However, in Cross 2, the coefficient of variation of F_2_ was the highest (2.09%) among all generations, whiles coefficients of variation for P_1_, P_2_, and F_1_ were 1%, 0.03% and 1.27% respectively (Table 4). Test for normality for the F_2_ segregants and backcross populations did not show a normal distribution. A Shapiro–Wilk test yielded *p*-values (two-tailed) of 0.011 and 0.002 for an alpha value of 0.05 for crosses 1 and 2. The Jarque-Bera test for normality, however, gave a contrary result (0.442 and 0.314) for an alpha value of 0.05 for F_2_ populations of crosses 1 and 2 respectively. Assuming the absence of normality, clear phenotypic classes may be established. Using a cooking time classification of short (up to 39 min), medium-cooking (40–59 min), long or slow-cooking (60–79 min), very or extra-long or extra slow-cooking (80 min and beyond) three arbitrary phenotypic classes can be created for the F_2_ populations of crosses 1 and 2. These have ratios 1:3:1 and 1:2:7 for F_2_ populations obtained for crosses 1 and 2 respectively. However, these phenotypic ratios do not fit principles of qualitative inheritance.

**TABLE 2:** Analysis of variance for cooking time in crosses 1 and 2.

	Source of variation	d.f.	Mean squares	*P*-value
**Cross 1**	Generations	5	987.64	<0.0001
**Cross 2**	Generations	5	1537.25	<0.0001

**TABLE 3:** Population mean distribution for cooking time among six basic generations derived from cross 1 [CRI-11(1)-1 × C9P(B)].

Generations	Means (minutes)	Standard errors	Coefficients of variation (%)
P_1_	24.00	±0.10	1.00
P_2_	71.30	±0.45	0.45
F_1_	58.00	±0.22	2.40
F_2_	48.00	±0.06	1.90
BC_1_P_1_	43.00	±0.08	2.00
BC_1_P_2_	52.53	±0.08	1.72

**TABLE 4:** Population mean distribution for cooking time among six basic generations derived from the cross 2 [CRI-11(1)-1 × TVu7687].

Generations	Means (minutes)	Standard errors	Coefficients of variation (%)
P_1_	24.00	± 0.10	1.00
P_3_	105.00	± 0.07	0.31
F_1_	67.00	±0.14	1.27
F_2_	65.45	± 0.09	2.07
BC_1_P_1_	46.00	± 0.05	1.16
BC_1_P_3_	77.48	± 0.05	0.75

Mean values, variances and their standard errors for the analyzed traits of the two crosses are presented (Tables 5, 6). Additive and dominance gene effects were found to be significant at *p* = 0.001. In both crosses (1 and 2 respectively), means of dominant gene effect were higher (−103.00 and −67.65) than the additive dominant gene effect (−33.35 and −38.93). Furthermore, both dominance and additive gene effects were found to be negative.

**TABLE 5:** Estimates of means, variances, and standard errors of gene effects for cross 1[CRI-11(1)-1 × C9P(B)].

Genetic components	Means	Variances	Standard error	Calculated *‘t’* value	*P*-value
[m]	71.00	10.125	± 3.18	22.30	0.001
[a]	−33.35	11.345	± 3.37	–9.90	0.001
[d]	−103.00	208.89	± 14.45	–7.10	0.001
[aa]	−106.3	207.38	± 14.40	–7.38	0.001
[ad]	10.15	11.41	± 3.38	3.00	0.01
[dd]	144.1	349.56	± 18.70	7.71	0.001

*[m], mean; [a], additive effect; [d], dominance effect; [aa], additive × additive; [ad], additive × dominant; and [dd], dominant × dominant.*

**TABLE 6:** Estimates of means, variances, and standard errors of gene effects for cross 2 [CRI-11(1)-1 × TVu7687].

Genetic components	Means	Variances	Standard error	Calculated ‘*t*’ value	*P*-value
[m]	70.25	6.48	±2.55	27.60	0.001
[a]	−38.93	7.55	±2.75	–14.17	0.001
[d]	−67.65	133.95	±11.57	–5.85	0.001
[aa]	−73.65	133.87	±11.57	–6.37	0.001
[ad]	1.58	7.59	±2.75	0.57	0.01
[dd]	75.1	224.76	±14.99	5.01	0.001

*[m], mean; [a], additive effect; [d], dominance effect; [aa], additive × additive; [ad], additive × dominant; and [dd], dominant × dominant.*

Significant epistatic additive × additive, additive × dominant and dominant × dominant gene effects were detected for cooking time for cross 1 [CRI-11(1)-1 × C9P(B)]. Mean value for the epistatic dominant × dominant gene effect was the highest among the additive and dominant gene effects as well as among the epistatic gene effects. The mean value for the epistatic additive × additive gene effect was found to be the second highest among the epistatic gene effects and yet higher than the additive and dominant gene effects. Epistatic additive × dominant gene effect was found to have lowest mean value and was the only gene effect found to be significant at *p* = 0.01. Additive and dominant gene effects were both negative in direction. In addition, the additive × additive gene effect was the only epistatic gene effect found to be negative. Similar results were obtained for cross 2 [CRI-11(1)-1 × TVu7687] are presented in Table 6. Significant epistatic additive × additive and dominant × dominant gene effects were detected for cooking time for this cross. Epistatic additive × dominant gene effect was the only gene effect detected to be non-significant. All other gene effects were significant at *p* = 0.001. Similarly, dominant gene effect was higher than additive gene effect. In addition, epistatic dominant × dominant gene effect was also higher than the mean value for additive × additive gene effect. Similar to cross 1 [CRI-11(1)-1 × C9P(B)], the additive and dominant gene effects were found to be negative. Furthermore, the epistatic additive × additive gene effect was the only epistatic gene effect found to be negative.

All gene effects were found to be significant for both crosses except epistatic additive × dominant gene effect in cross 2 [CRI-11(1)-1 × TVu7687]. In addition, epistatic dominance × dominance, epistatic additive × dominance, dominance and additive gene effects were values in decreasing order of magnitude in both crosses. Furthermore, epistatic additive × dominance, dominance and additive gene effects were found to be negative. All remaining epistatic gene effects were found to be positive.

### Heritability Estimates

Broad sense and narrow sense heritabilities were computed for each cross and presented in Table 7. Broad sense heritabilities obtained were 0.94 and 0.99 for crosses 1 and 2 respectively. Broad sense heritabilities for the two crosses were generally high but marginally higher for cross 2 than for cross 1. Also, high narrow sense heritabilities of 0.88 and 0.84 were obtained for crosses 1 and 2 respectively, although the narrow sense heritability of cross 1 was marginally higher than cross 2. Generally, narrow sense heritabilities were lower than broad sense heritabilities for both crosses. Degree of dominance was higher (−0.15) in cross 2 than for cross 1 (0.08a). Thus, in both crosses, the degree of dominance was between zero and one (−1 < D < 0) for cooking time.

**TABLE 7:** Estimates of broad sense and narrow sense heritabilities in two crosses in cowpea.

Cross	Parameter	Estimate
1 [11(1)-1 × C9P(B)]	Broad sense heritability	0.94
	Narrow sense heritability	0.88
	Degree of dominance (a)	–0.08
2 [11(1)-1 × TVu7687]	Broad sense heritability	0.99
	Narrow sense heritability	0.84
	Degree of dominance (a)	–0.15

### Phenotypic and Genotypic Coefficient of Variation and Genetic Advance

Variances, coefficient of variability and genetic advance of cooking time (after storage) in two crosses in cowpea are presented (Table 8). In both crosses, the differences between the genotypic and phenotypic coefficient of variation values were marginal. Genetic advance for cross 1 was 27.09 which was lower than the value obtained for cross 2 (40.4).

**TABLE 8:** Variances, coefficient of variability and genetic advance of cooking time (after storage) in two crosses in cowpea.

Parameter	Denotation	Cross 1 [11(1)-1 × C9P(B)]	Cross 2 [11(1)-1 × TVu7687]
Mean	μ	48.70	67.73
Phenotypic variation	δ^2^p	195.63	392.35
Genotypic variance	δ^2^g	194.13	389.86
Phenotypic coefficient of variation	PCV (%)	28.73	29.25
Genotypic coefficient of variation	GCV (%)	28.60	29.15
Broad sense heritability	$h_{\text{b}}^{2}$	0.94	0.99
Genetic advance	GA	27.09	40.4

## Discussion

### Genetic Variability and Gene Effects

The differences among analyzed generations were sufficient to perform generation mean analysis for both crosses. In both crosses, the means of F_1_ fell between cooking time values for both parents indicating some level of partial or incomplete dominance. Observed variations among populations of both crosses indicate a high possibility of response to selection for cooking time trait. It also indicates that parents chosen for these bi-parental crosses, to a large extent, had the contrasting expressions for the cooking time trait. The extent of variation among populations is useful since the progress of any breeding program depends largely on the existence of genetic variability within the population available (Akhshi et al., 2014). Population mean values obtained for both crosses which showed that P_1_, BC_1_P_1_, and F_1_ were skewed toward the short cooking time parent indicate dominance over long-cooking time although other gene effects could play a role in the inheritance of cooking time. Elia (2003) attributed skewness of the distribution in cooking time in favor of short cooking parents to either maternal effects, or control by dominant genes.

Relatively higher coefficients of variations observed for especially the segregating generations such as F_2_ and the backcrosses, can be attributed to the combined action and interaction of various gene effects as well as the environment. Coefficients of variation indicate the level of dispersion around the mean. Consequently, the higher the coefficient of variation, the greater the level of dispersion around the mean. On the other hand, the relatively lower coefficients of variation for cooking time values of parents (P_1_, P_2_, and P_3_) is attributed mainly to effect of environmental factors (Acquaah, 2007). This is because parents are considered homozygous while F_1_ progenies are uniformly heterozygous. This phenomenon generally indicates that cooking time trait in cowpeas is quantitatively inherited. In this study, results of the Shapiro–Wilk test for normality suggest the occurrence of qualitative inheritance of the cooking time. However, ratios of computed phenotypic classes did not fit any expected Mendelian ratios for qualitative inheritance. Jarque–Bera test for normality for the F_2_ populations for both crosses however showed continuous distribution indicating the occurrence of quantitative inheritance. In an evaluation of cooking time on 140 F2:4 families segregating for cooking time, six QTL were identified for cooking time in common beans in a single environment (Garcia et al., 2012), In another study in common beans, Cichy et al. (2015) also found markers with small effects on cooking time. They found regions on chromosomes Pv02, Pv03, and Pv06 associated with cooking time.

Furthermore, ratios obtained for both crosses were not similar. This difference may be due to the brown-seeded parent (TVu7687) involved in the Cross 2. Generally, colored cowpeas are known to be long-cooking while white or cream-colored genotypes are fast-cooking (Addy et al., 2016). According to Cichy et al. (2015), three QTL on chromosome Pv06 which were associated with cooking time had locations also associated with three UDP-glucosyl transferase genes. This gene family is reported to be involved in flavonoid biosynthesis and the development of pigments (Bowles et al., 2005). Seed coat color due to the presence of anthocyanins was correlated with cooking time.

Significant but negative additive gene effects obtained for both crosses is an indication of assurance of gains with selection for fast-cooking trait in the long term (Acquaah, 2007). The negative value of the dominance gene effect, observed showed that the alleles responsible for less value of the trait (fast or fast-cooking) was dominant over the alleles controlling high value of the trait (long or slow-cooking). This result is in agreement with the work of Jacinto-Hernandez et al. (2003) who reported on the dominance of short cooking time in *Phaseolus vulgaris* L. in Mexico. Negative values for the additive gene effect indicates that alleles conferring short cooking time and not for the long-cooking time trait are responsible for parent-offspring resemblance. The existence of significant and negative additive gene effect would make selection for short cooking time at early stages of breeding effective. However, due to significant epistatic dominance × dominance, epistatic additive × dominance gene effects in cross [CRI-11(1)-1 × C9P(B)] and significant epistatic dominance × dominance gene effect observed in cross [CRI-11(1)-1 × TVu7687] selection for short cooking time trait at advanced generations would be the effective breeding decision to consider.

### Heritability Estimates

High estimates for broad and narrow sense heritabilities obtained for storage-induced cooking time are similar to values reported from other studies. Nielsen et al. (1993) reported broad sense heritability of 0.76 and a narrow sense heritability ranging from 58 to 85% for cooking time in cowpea. Investigations using lentils gave a broad sense heritability of 0.82 for cooking time (Erskine et al., 1985). Elia et al. (1997) also reported a high value of 0.9 narrow sense heritability for the same trait in *Phaseolus vulgaris* in Tanzania while Jacinto-Hernandez et al. (2003) reported a value of 0.74 narrow sense heritability for the same trait in *Phaseolus vulgaris* in Mexico. In a dry bean GxE study across nine locations in Africa, North America, and the Caribbean, the heritability of cooking time was 98% on presoaked beans and 60% on unsoaked beans (Cichy et al., 2019). Mashi (2006) also reported estimates of heritability in the broad sense ranged from 89 to 95% and a range of 58–85% for narrow sense heritability in cowpeas. Generally, both broad and narrow sense heritabilities reported are high for both cowpeas and beans. Similar range of heritability estimates between cowpeas and beans is probable because they are closely related species. These high estimates of both broad and narrow sense heritabilities obtained in this study agree with the generally high estimates reported for both cowpeas and beans. According to Stansfield (1988), traits with narrow sense heritability higher than 50% are considered to have a high heritability. High heritability estimates may be due to high additive genetic variance in relation to the total phenotypic variance for the trait. High narrow sense heritability indicates a high measure of the predictability of offspring trait values that is based on parental trait values. This means it is possible to make genetic advancements for the cooking time trait. Although, heritability estimates reported and obtained from this study are within similar ranges, there is need for some clarity in the type of cooking time examined by these authors. These authors may have worked on cowpeas or beans grains at different storage periods and conditions. This is due to the trigering and increasing effect of the ‘hard-to-cook’ phenomenon or grain hardening during storage. This is a phenomenon, typical of cowpeas and beans (Coelho et al., 2007), and occurs during storage in environmental conditions such as high temperatures (30–40°C) and high humidity (>75%) prevalent in the tropics and sub-tropics. A clear distinction, therefore, needs to be made for data of cooking time obtained in any study of this nature. This study specifically examined the heritability of storage-induced cooking time in cowpeas. It is likely that some or all studies on cooking time may have been carried out with seeds that had the hard-to-cook phenomenon already triggered.

The degree of dominance was between zero and one (−1 < D < 0) for cooking time indicating incomplete or partial dominance with marginal magnitude in the direction of the long cooking parent. In addition, mid-parent values were lower than F_1_ means for cooking time in the two crosses. It indicates that that the cooking time of a heterozygous genotype is distinct from and often intermediate to the phenotypes of the homozygous genotypes. However, the degree of partial dominance obtained for both crosses were skewed toward zero than −1. It follows also that for the two crosses studied the means of cooking time of the first filial generation were toward the fast-cooking trait than for the long-cooking trait.

### Phenotypic and Genotypic Coefficient of Variation and Genetic Advance

Estimates of degree of dominance between zero and one indicate low effect of environment on the expression of cooking time in cowpeas. Low to moderate coefficients of variation of phenotype and genotype indicate low to moderate influence of environment on the trait thus making it a reliable one for selection. However, high genetic coefficient of variation recorded for cooking time alone is not sufficient for the determination of the extent of the advance to be expected by selection. Burton (1952) suggested that genetic coefficient of variation together with heritability estimates would give the best picture of the extent of the advance to be expected by selection. Considering heritability estimates in conjunction with the predicted genetic gain constitute a more reliable strategy for selection. Although heritability estimates are the true indicators of potential genetic ability of genotypes that can be used as a tool for selection, changes in the values of the heritability estimates due to changes in the environment suggest that total dependence on such estimates should be done with a lot of caution. Therefore, the high heritability estimates and moderate to high genetic advance for the fast-cooking trait in the present study indicate that it is controlled by additive gene action rendering phenotypic selection for cooking time an effective strategy (Tazeen et al., 2009). This means that the additive nature of genetic variation was transmitted from the parents to the progeny.

## Conclusion

Genetic studies on storage-induced cooking time trait in cowpea have rarely been reported previously. This study showed that fast-cooking time was dominant over long-cooking time. High broad and narrow sense heritabilities with moderate genetic advances indicated the additive nature of inheritance. However, due to significant epistatic gene effects observed, selection for fast-cooking time trait at advanced generations would be the effective breeding decision to consider. This is so especially when cooking time evaluation is time consuming and somewhat laborious. Finally, since evaluation of cooking time in cowpea is expensive and time consuming, we recommend that additional studies be carried out to identify molecular markers for the fast-cooking trait as a means for indirect selection. The development of fast-cooking cowpea varieties acceptable to consumers have the potential to increase their per capita consumption and help reduce the emission of greenhouse gasses through prolonged burning of fuel during cooking.

## Data Availability Statement

The datasets generated for this study are available on request to the corresponding author.

## Author Contributions

SA initiated and designed the research, conducted the research, analyzed the data, and had primary responsibility for final content. SA and KC provided essential materials and workspace. SA, KC, HA-D, IA, SO, and AE wrote the manuscript. All authors have read and approved the final manuscript.

## Conflict of Interest

The authors declare that the research was conducted in the absence of any commercial or financial relationships that could be construed as a potential conflict of interest.
